# Case Report: Ileocecal Intussusception Secondary to a Metastatic Malignant Melanoma From the Scalp

**DOI:** 10.7759/cureus.68191

**Published:** 2024-08-30

**Authors:** Yukai Chen, Zhongjie Liu, Yu Wu, Xiangxi Zhu, Honglei Wang

**Affiliations:** 1 Graduate School, Tianjin Medical University, Tianjin, CHN; 2 Department of Gastrointestinal Surgery, Tianjin Hospital of ITCWM Nankai Hospital, Tianjin, CHN; 3 Key Laboratory of Acute Abdomen Disease Associated Organ Injury and ITCWM Repair, Tianjin Hospital of ITCWM Nankai Hospital, Tianjin, CHN; 4 Tianjin Institute of Integrative Medicine for Acute Abdominal Diseases, Tianjin Hospital of ITCWM Nankai Hospital, Tianjin, CHN; 5 Department of Clinical Medicine, Zunyi Medical University, Zhuhai, CHN

**Keywords:** intestinal metastasis, malignant melanoma, case report, small bowel resection, ileocecal intussusception

## Abstract

Adult intussusception is an unusual condition that is infrequently associated with malignancy. We experienced a rare occurrence of ileocecal intussusception in a 31-year-old male, secondary to metastatic melanoma. The patient, with a medical history of scalp malignant melanoma treated surgically and with chemotherapy, presented with persistent lower abdominal pain. Diagnostic modalities, including computed tomography (CT) and colonoscopy, revealed ileocecal intussusception. Subsequent laparoscopic surgery and pathological examination identified metastatic melanoma as the etiological factor for the intussusception, leading to the surgical resection of the involved bowel segment. The postoperative period was marked by a smooth recovery with no complications. This case shows the importance of considering malignancy in the differential diagnosis of adult intussusception and underscores the urgency of surgical intervention in the management of such cases.

## Introduction

Malignant melanoma is one of the rarest forms of skin malignancy, frequently occurring in areas such as the skin, orbit, oral cavity, and nasal mucosa. Malignant melanoma is known for its strong metastatic capability [1], with common sites of dissemination including the gastrointestinal tract and liver, among which the small intestine, due to its rich blood supply, accounts for the most frequent site of gastrointestinal metastases (71%-91%), followed by the colon (22%-26%) [2].

For adults, intussusception is a rare acute abdominal disease that usually has a clear cause, such as a malignancy. It is one of the common clinical presentations of intestinal metastasis from malignant melanoma, characterized by its abrupt onset and nonspecific symptoms, lacking distinctive diagnostic tests. It frequently requires emergency surgical intervention even when the diagnosis is not yet clear. We experienced a case of ileocecal intussusception secondary to small intestinal metastasis from malignant melanoma and discusses its clinical features, providing a reference for clinical practice.

## Case presentation

A 31-year-old male presented with intermittent lower abdominal pain for five months, with a worsening over the past one day. The patient had experienced intermittent lower abdominal pain over the previous five months, which he had not attended to at the time. One day before admission, the patient experienced sudden severe lower abdominal pain and went to a local hospital. An abdominal computed tomography (CT) scan suggested "abnormal intestinal density in the right abdomen, considering intussusception." Pain relief and intravenous nutrition were administered but did not significantly alleviate the symptoms. The patient had a history of malignant melanoma of the scalp for which he had undergone surgical resection a year prior, followed by 12 cycles of chemotherapy, the specifics of which are not detailed. The patient had no family history of tumors.

**Figure 1:**
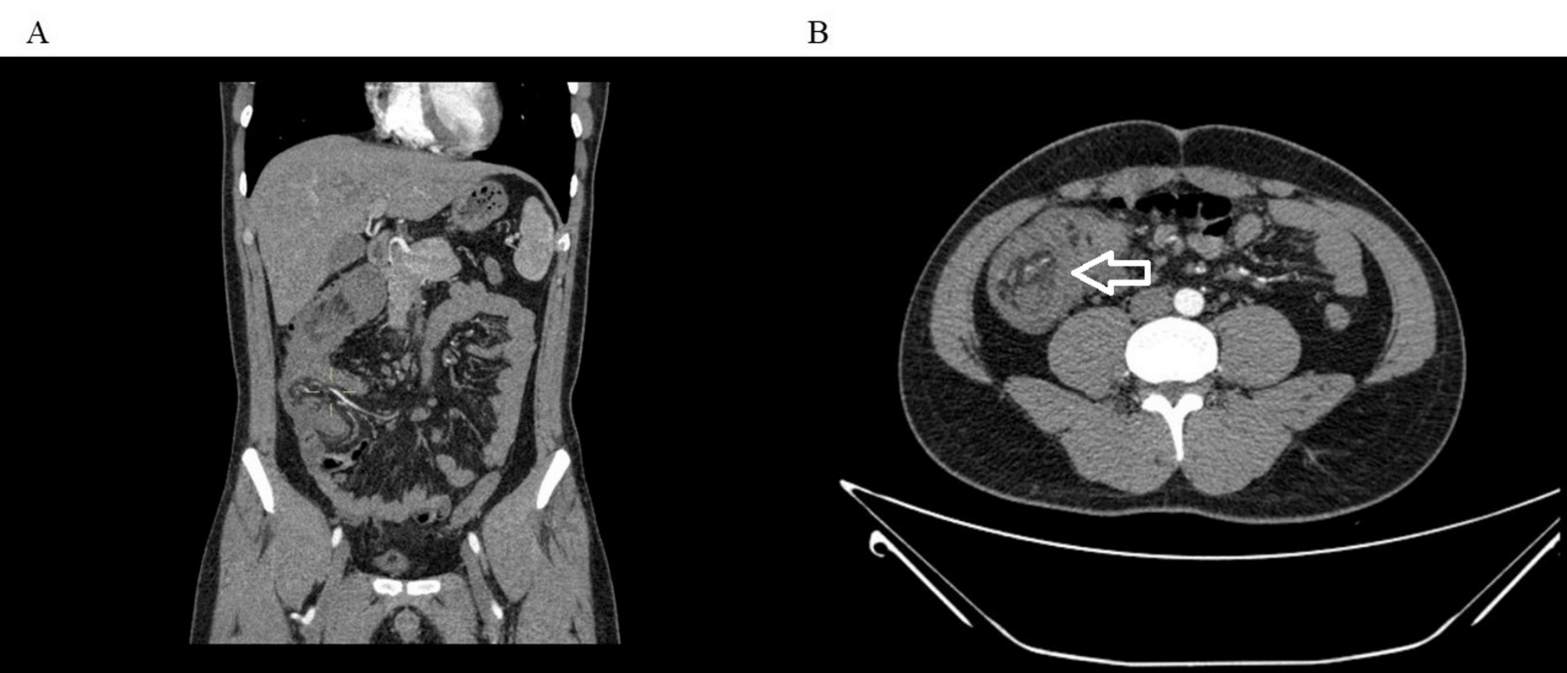
Enhanced abdominal CT imaging. (A) Coronal plane showing the terminal ileum and mesentery entering the cecum (yellow cross). (B) Axial plane demonstrating the "target sign" CT: Computed tomography

**Figure 2:**
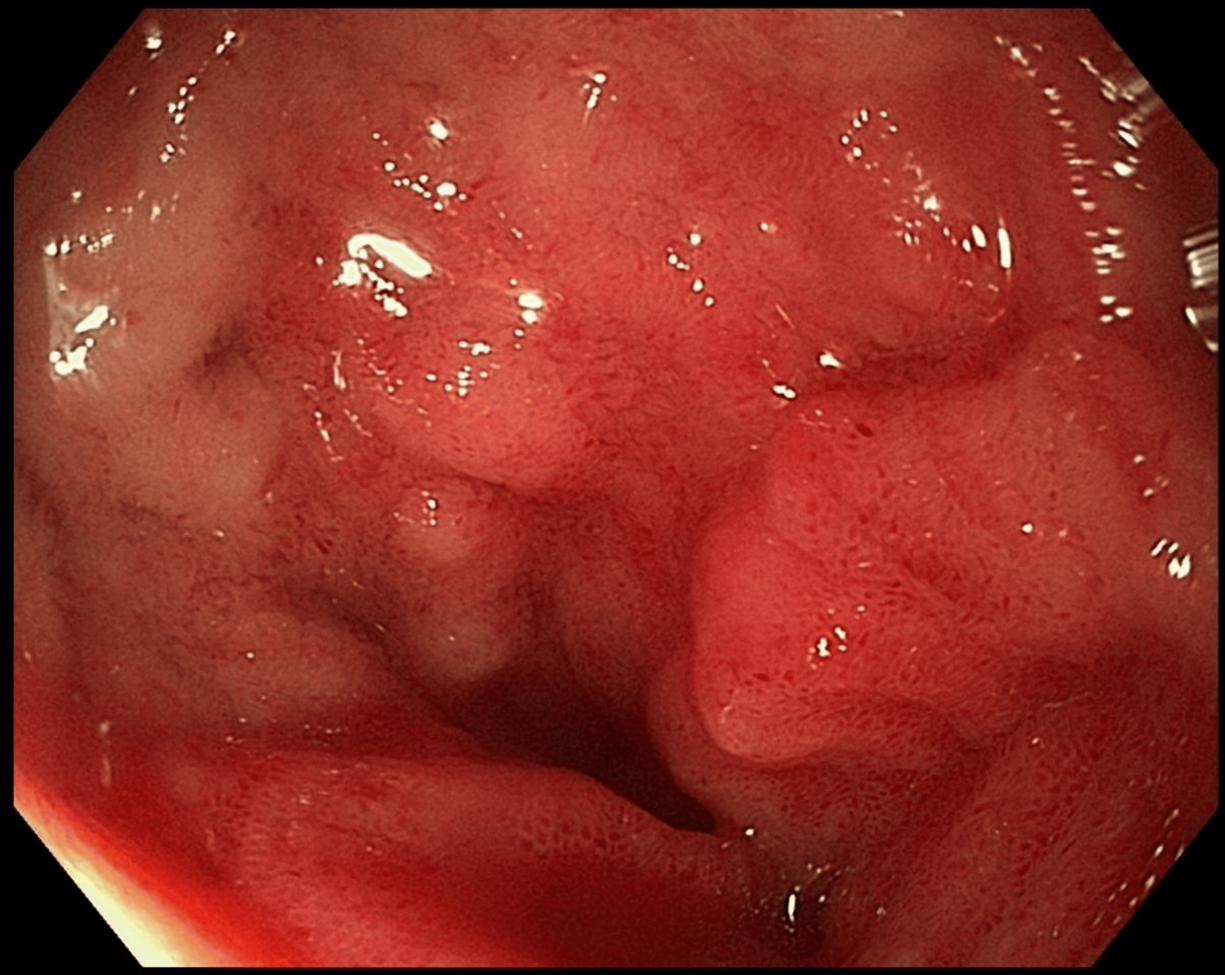
Colonoscopy revealing mucosal lesions in the terminal ileum

Physical examination upon admission revealed tenderness in the lower right abdomen with a palpable mass approximately 4 cm in size, which was mobile; bowel sounds were active, with no other remarkable physical signs. Laboratory tests upon admission indicated mild anemia with hemoglobin at 91 g/L and hematocrit (Hct) at 29.1%. Additionally, he had mild renal dysfunction with serum creatinine at 109 umol/L and uric acid at 538 umol/L. The patient sought further treatment at Tianjin Nankai Hospital, where he was admitted through the emergency department with intussusception on May 8, 2024. An enhanced abdominal CT scan was performed on May 11. As is seen in Figure 1, the CT scan showed thickened terminal ileum and ileocecal valve walls, with part of the terminal ileum structure and mesentery entering the nearby colon, presenting a "target sign," and thickening of the ascending colon wall, suggesting the presence of intussusception. Colonoscopy only revealed mucosal lesions in the terminal ileum and did not show any definitive intussusception (Figure 2).

**Figure 3:**
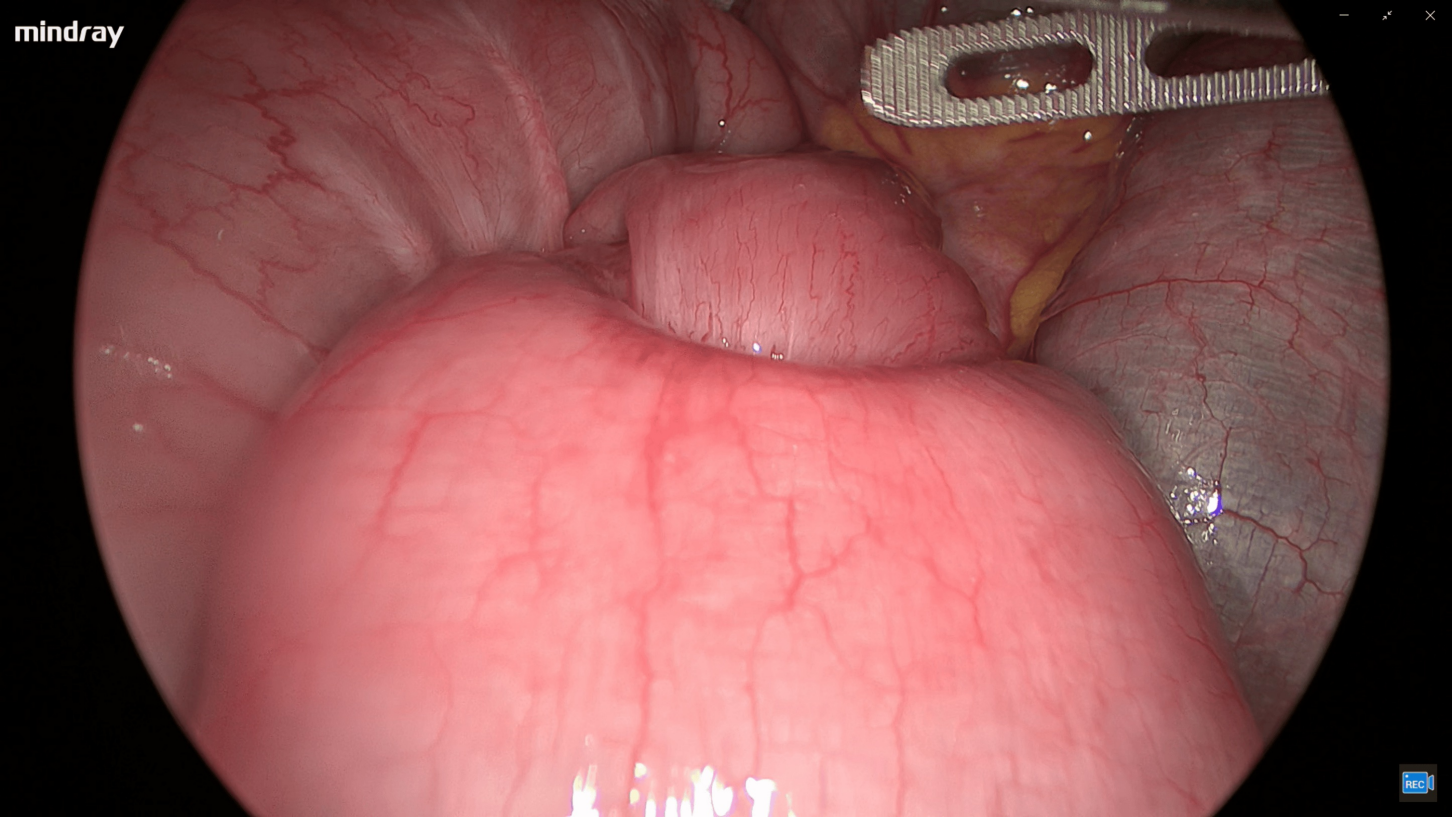
Intraoperative laparoscopic view of the tumor site

After admission, the patient was put on fasting and fluid therapy for five days. However, the patient's abdominal pain had not relieved. After another enhanced abdominal CT scan that confirmed the diagnosis of ileocecal intussusception, a diagnostic laparoscopy was conducted the next day. During the operation, it was found that the intussusception involving the terminal ileum and cecum had resolved. The tumor was located 60 cm from the ileocecal valve with mild proximal bowel dilation, as seen in Figure 3 under laparoscopy. No other tumors were found in the abdominal cavity, and a partial small intestine resection and anastomosis were performed under laparoscopy.

Histopathological examination showed a raised lesion measuring approximately 3.1 * 2.5 * 0.9 cm, mainly located in the submucosal muscle layer, extending to the mucosal layer. Four peri-intestinal lymph nodes were examined without evidence of tumor involvement. Immunohistochemistry included the following: S100 (+), MelanA (+), HMB45 (+), Ki67 (30%+), CKpan (-), vimentin (+), CD117 (weak+), Dog-1 (-), CD34 (-), and P53 (weak+).

The final diagnosis was malignant melanoma of the small intestine, clinically considered metastatic. The patient recovered well postoperatively and was discharged on the 10th postoperative day (POD10). This case report follows the CARE (CAse REports) Guidelines. The timeline of the presented case is shown in Table 1.

**Table 1:** Timeline of the presented case CT: Computed tomography

Date	Treatment
One year before initial presentation	Resection of melanoma on the scalp, followed by 12 cycles of chemotherapy
Five months before initial presentation: Intermittent lower abdominal pain	Without paying attention
One day before initial presentation: Sudden onset of severe lower abdominal pain	Presented at a local hospital, where abdominal CT suggested intussusception; conservative treatment was administered without relief
May 8, 2024: Persistent lower abdominal pain	Visited our emergency department for further treatments
May 13, 2024: Abdominal pain not relieved	Enhanced CT scan confirmed intussusception
May 14, 2024: Preoperative examinations completed	Underwent small bowel resection, anastomosis, and abdominal cavity drainage were performed under laparoscopy
May 18, 2024: Postoperative day (POD4)	Histopathological examination completed. Malignant melanoma of the small intestine was confirmed
May 24, 2024 (POD10): Demonstrated good postoperative recovery	Discharged from the hospital

## Discussion

Compared to other malignant tumors, malignant melanoma exhibits greater invasiveness and a strong propensity for metastasis. Beyond common sites of metastasis such as the liver and lungs, malignant melanoma has a predilection for gastrointestinal tract invasion. While symptomatic gastrointestinal involvement occurs in 1%-5% of patients with malignant melanoma, autopsy studies reveal that up to 60% of patients demonstrate gastrointestinal tract metastasis [3], which may be associated with atypical clinical symptoms and a high rate of misdiagnosis.

Adult intussusception is an uncommon condition which accounts for 1%-5% of patients having bowel obstruction, and 5%-10% of all intussusception cases [4,5]. Unlike intussusception in children that is often idiopathic, adult intussusception is often secondary to a definable cause, such as tumors, polyps, or diverticula, especially in the case of ileocecal intussusception. Studies indicate that approximately 50% of small bowel intussusceptions caused by metastatic malignancies were due to metastatic melanoma [6]. Intussusception can be categorized into four types based on the involved segment: enteric-enteric, ileocecal, colonic-colonic, and sigmoidorectal, with ileocecal intussusception accounting for approximately 29.1%-52.9% of all adult cases [7,8].

The clinical symptoms of adult intussusception are atypical and often recurrent, including abdominal pain, distension, nausea, vomiting, changes in bowel habits, and lower gastrointestinal bleeding [8]. In this case, the patient presented with persistent abdominal pain over several months, ultimately leading to a diagnosis of intussusception due to the exacerbation of pain.

For diagnosis, adult intussusception typically follows a sequence of confirming intussusception, localization, and endoscopic biopsy. Abdominal CT is essential in preoperative diagnosis and evaluation, displaying the length and diameter of intussusception, the three-dimensional view of the intestine and surrounding organs, the starting point, type, and position of intussusception, mesenteric vascular system, the possibility of strangulation, and the possibility of partial or complete intestinal obstruction [9]. It is also a sensitive modality for identifying adult intussusception, with a diagnostic accuracy of 58%-100% [8]. Colonoscopy allows direct visualization of the intussuscepted bowel segment and biopsy to identify the cause, but its utility is limited to the colon and the terminal ileum near the ileocecal area, thus serving as a supplementary tool.

Given that adult intussusception is often induced by malignant tumors, surgical resection is the preferred treatment option. In particular, laparoscopic surgery is considered a safe alternative when the preoperative diagnosis is unclear [10]. In this case, two abdominal CT scans and one colonoscopy failed to clarify the cause of the intussusception. However, based on the definitive diagnosis of intussusception and a history of malignant melanoma, the possibility of intussusception due to malignancy could not be excluded preoperatively. Therefore, we decided to proceed with laparoscopic surgery, during which the presence of metastatic melanoma was confirmed. Ultimately, the metastatic lesion was resected to reduce the patient's tumor burden.

Large-sample studies suggest that melanoma of the head and neck has a worse prognosis compared to trunk melanoma, with a higher risk of all-cause mortality [11]. The prognosis worsens if metastasis occurs, with a median survival of 21.1 months [12]. The patient in this case underwent resection for scalp malignant melanoma a year prior to admission and received regular chemotherapy, later presenting with abdominal pain and intussusception, which led to potentially life-saving treatment. The overall survival rate for patients with metastatic melanoma who undergo complete resection of gastrointestinal metastases is significantly improved, with a median survival period of 64 months [13]. In contrast, patients who opt for palliative care and nonsurgical interventions have a median life expectancy of only seven months [13]. This significant difference underscores the substantial survival benefit that surgical resection can offer to patients with gastrointestinal metastases from melanoma. However, it should be acknowledged that surgical resection still carries potential risks and limitations, including incomplete resection, tumor cell dissemination, anastomotic complications, and postoperative infection [8]. Therefore, to optimize treatment strategies, a comprehensive individualized assessment of each patient, such as the condition, overall status, and feasibility of complete resection, remains necessary.

## Conclusions

In conclusion, this case report substantiates the critical need for a high index of suspicion for malignancy in adults presenting with intussusception, particularly in patients with a history of melanoma. Laparoscopic surgery, as a minimally invasive approach, should be considered when the cause of intussusception is unclear. From this case, surgeons can glean the importance of integrating radiological and endoscopic findings with clinical history to expedite surgical intervention, which can significantly improve patient outcomes. The nonspecific symptoms and the efficacy of surgical resection in such cases should be further investigated to refine management strategies and improve the survival of patients.
